# The Otolaryngology boot camp: a scoping review evaluating commonalities and appraisal for curriculum design and delivery

**DOI:** 10.1186/s40463-022-00583-9

**Published:** 2022-06-04

**Authors:** Adom Bondzi-Simpson, C. J. Lindo, Monica Hoy, Justin T. Lui

**Affiliations:** 1 Temerty Faculty of Medicine, University of Toronto, Toronto, ON Canada; 2 Division of General Surgery, Department of Surgery, University of Toronto, Toronto, ON Canada; 3 Section of Otolaryngology–Head and Neck Surgery, Department of Surgery, University of Calgary, Calgary, AB Canada

**Keywords:** Boot camp, Training course, Surgical education, Medical education, Otolaryngology, Surgical training

## Abstract

**Objective:**

Surgical boot camps are becoming increasingly popular in Otolaryngology–Head and Neck Surgery (OHNS) residency programs. Despite pioneering virtual reality and simulation-based surgical education, these boot camps have lacked critical appraisal. The objective of this article was to examine the adoption and utility of surgical boot camps in OHNS residency training programs around the world.

**Data Sources:**

Ovid Medline and PubMed databases were systematically searched in accordance with the Preferred Reporting Items for Systematic Reviews and Meta-Analyses (PRISMA) guidelines for scoping reviews. Additionally, a grey literature search was performed.

**Review Methods:**

Inclusion criteria were peer-reviewed publications and grey literature sources that reported on OHNS boot camps for the novice learner. The search was restricted to human studies published in English. Studies were excluded if they were not examining junior trainees.

**Results:**

A total of 551 articles were identified**.** Following removal of duplicates, screening, and full text review, 16 articles were included for analysis. Seven major boot camps were identified across various academic sites in the world. Most boot camps were one-day intensive camps incorporating a mixture of didactic, skill specific, and simulation sessions using an array of task trainers and high-fidelity simulators focusing on OHNS emergencies. Studies measuring trainee outcomes demonstrated improvement in trainee confidence, immediate knowledge, and skill acquisition.

**Conclusion:**

Surgical boot camps appear to be an effective tool for short term knowledge and skill acquisition. Further studies should examine retention of skill and maintenance of confidence over longer intervals, as little is known about these lasting effects.

## Introduction

Upon completing medical school, junior trainees enter post-graduate training programs with dramatically increased responsibilities. To address the concern regarding trainee skill inadequacy, surgical boot camps were developed to help develop skillsets from interpreting diagnostic imaging to performing surgical procedures [1].

The educational design of most surgical boot camps is a combination of didactic learning and small group simulation sessions. Both governing medical educational bodies of Canada (Royal College of Physicians and Surgeons of Canada) and the United States (Accreditation Council for Graduate Medical Education) have embraced competency-based educational frameworks for post graduate medical education (PGME) [2]. These frameworks are an outcomes-based approach to curriculum design where trainee advancement is dependent on mastering entrustable professional activities (EPA’s) [1]. With this shift, simulation training is integral in allowing trainees to practice clinical and procedural skills in areas specifically identified as key competencies or milestones before encountering real patient scenarios [1].

Literature examining the role of surgical boot camps has been extensively covered over the past decade. The majority of studies have examined the following outcomes: knowledge and technical skills acquisition, team communication skill development, and individual confidence improvement [3–6]. Moreover, surgical boot camps allow for social and cultural welcoming [7, 8]. Despite widespread adoption by various surgical specialties, including cardiac, general, neuro, orthopedic, trauma, and vascular surgery, few surgical boot camps have been reported on in Otolaryngology–Head and Neck Surgery (OHNS) [9–16]. Furthermore, OHNS boot camps lack critical appraisal despite being one of the leaders in virtual reality and simulation-based surgical education [17, 18]. The goal of this scoping review was to examine the utility of PGME surgical boot camps in OHNS around the world. To achieve this goal, this manuscript will address four fundamental objectives. (1) Thoroughly summarize existing OHNS boot camps around the world. (2) Determine overlap in curriculum design and delivery, resources, and simulation. (3) Examine pros and cons of existing boot camp formats. (4) Suggest an optimal boot camp design for junior residents in OHNS.

## Methods

A scoping review based on the Preferred Reporting Items for Systematic Reviews and Meta-Analysis scoping review (PRISMA-ScR) guidelines was performed in February 2021 [19]. The research databases included were Ovid Medline (September 1946 – February 2021) and PubMed (January 1946 –February 2021). The search terms included [(otolaryngology/otorhinolaryngology/ear nose throat/ENT/ORL/head and neck surgery) AND (“boot camp/bootcamp/training course)]. Inclusion criteria were peer-reviewed publications comparing pre- and post-course quantitative and qualitative data in skill performance or knowledge acquisition. The search was restricted to human studies published in English. In addition to the peer-reviewed search, an online grey literature search was utilized, specifically looking at conference proceedings and published information from medical educational and department websites. Excluded studies were non-English publications and studies not examining OHNS interns or junior residents (PGY-1 and PGY-2). Due to boot camps typically being introductory camps, the search was limited to junior residents. All other articles including opinion pieces and editorials were included for qualitative analysis.

Four reviewers (A.B-S., C.J.L., M.Y.H., & J.T.L.,) independently screened all abstracts to identify studies that fulfilled the predetermined eligibility criteria. Any disagreement between the reviewers was resolved by consensus. Qualitative data from each included study was extracted using standardized data forms including the study’s title, author(s), year of publication, education themes, and outcomes assessed.

## Results

**Fig. 1:**
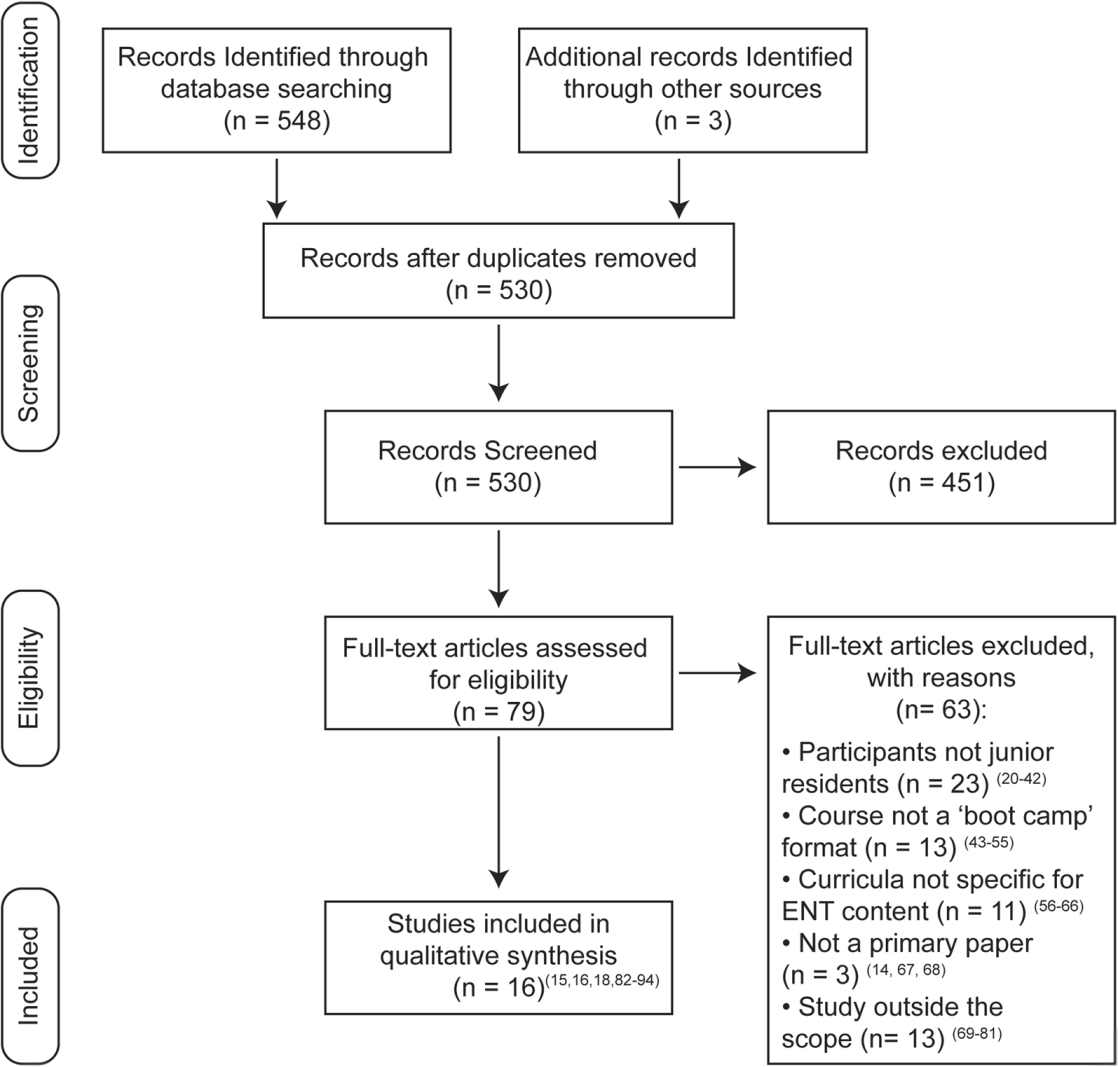
PRISMA flow diagram

A total of 21 articles were identified by Ovid Medline, 527 articles by PubMed, and 3 articles from a grey literature search. Following the removal of the duplicate records, 530 abstracts were screened (Fig. 1) [20–94]. Of the 79 articles that underwent full-text review, 63 articles were excluded, and the remaining 16 articles underwent complete qualitative analysis with the data being summarized in Tables 1, 2, 3, 4 and 5.

**Table 1:** OHNS boot camps publications

Study	Camp setting	Camp format	Outcomes assessed	Result
Malekzadeh et al. (2011)[87]	Georgetown University, USA	Cross-sectional study, one-day camp Six technical skills stations, telephone inquiry triage, and two complex airway scenarios	Confidence gained Perceived knowledge Technical skills Clinical performance measured immediately and at 6 months	Course was successful in improving immediate: knowledge, technical skills, and confidence up to 6 months post-course
Amin et al. (2013)[82]	New York University, USA	Prospective cohort study (6 months) Didactic lectures, cadaveric dissection, and simulations	Airway competencies using objective validated educational tools	Significant improvement in MCQ scores and faculty-based assessment of performance Hands on training most effective component
Zapanta et al. (2013)[94]	Georgetown University, USA & Western University, CAN	Qualitative phenomenological study Cross-sectional study, one-day camp	Resident learner experience	Residents’ goals are to increase knowledge Previous experience performing tasks and realism in camp scenarios influenced learning Developing teamwork/leadership valued Participants learn primarily through synthesis and application of knowledge
Chin et al. (2014)[15]	Western University, CAN	Cross-sectional study, one-day camp Seven technical skills stations, two high fidelity emergency scenarios, interactive panel discussion of 16 cases	Feasibility of course Perceived effectiveness of course relative to learning styles of residents	Majority of learning styles preferred active experimentation Residents highly value: variety, realism of simulation, and realism of task simulators 93% would recommend the program to their juniors
Malloy, Malekzadeh & Deutsch et al. (2014)[18, 86, 88]	Georgetown University, USA	Cross-sectional study, one-day camp Fundamental skills stations, special skills stations, two simulation scenarios, and interactive panel discussion	“How-to guide.”	Boot camps utilizing inter-institutional participants and faculty are effective
Bunting et al. (2015)[83]	Georgetown University, USA	Cross-sectional study, one-day camp	Realism and utility of novel PTA simulator	Participants believe PTA simulation is an effective teaching toll that would be useful for increasing competency before their first PTA drainage
Smith et al. (2015)[91]	Luton and Dunstable Hospital, Luton, UK & University of Cambridge, UK	Single-blinded, prospective RCT Cross-sectional study, one-day camp	Trainee’s perception of training and impact on performance Is a simulation-based OHNS emergencies camp superior to traditional lecture-based learning?	Participants in the simulation group rated training as “highly thought of,” and were more likely to recommend the teaching to a colleague versus those in the standard group A hybrid of lectures and simulation more effective for teaching OHNS emergency management than traditional lecture-based training
Scott et al. (2016)[90]	Western University, CAN	Cross-sectional study, one-day camp	Realism and utility a novel high-fidelity PTA simulator	Nearly 95% of participants were in strong agreement that objectives were met, and faculty members were effective for teaching 81% of participants agreed that the models were realistic and high quality 95% of OHNS faculty agreed the novel PTA simulator was representative of real life
Chin et al. (2016)[16]	Western University, CAN	Cross-sectional study, one-day camp	“How to guide.” Confidence performing routine OHNS emergency procedures, communication, teamwork, and stress handling skills before and after camp	Before camp participants had the most experience and confidence in intubation and bag mask ventilation and were least confident in managing retro-orbital hematomas After camp, there was a statistically significant increase in trainee confidence in 6 of the 10 procedures and confidence for triaging OHNS calls
Smith et al. (2016)[92]	University of Cambridge, UK	Cross-sectional study, one-day camp Focused lectures, practical skills training, emergency scenario simulation, and small group sessions	Feasibility of course for junior OHNS residents Knowledge of OHNS emergencies and perception of educational experience before and after camp	Statistically significant improvement on MCQ exam post-course 100% of trainees scored the boot camp as “highly thought of”. 84% of trainees would strongly recommend course 100% of trainees reported improvement in confidence performing OHNS exams and dealing with OHNS emergencies
Kiffel et al. (2017)[89]	Albert Einstein College of Medicine, New York, USA	Prospective cohort study, four-week curriculum 24 sessions divided into three categories: simulation, technical skills development, and didactic teaching	No outcomes were assessed	No results or conclusions were reported
Swords et al. (2017)[93]	Addenbrooke's Hospital, UK	Cross-sectional study, one-day camp Prospective, single-blinded design was used Focused lectures, small group sessions, practical skills training, and emergency scenario simulation	Acquisition of OHNS emergency skills	Immediate improvement in participant confidence that was maintained two to four months post course Blind assessment of performance during simulation sessions showed significant improvements across four key areas: diagnosis, systematic approach, airway breathing and circulation assessment, and ongoing management
Fuller et al. (2019)[85]	Hospital Un Canto a la Vida, Quito, EC	Three-day teaching course Prospective cohort study	Knowledge and skills in each of the targeted subject areas before and after the course The quality of each portion of the module Feedback on portions of the course that were enjoyable and those that were not	A statically significant increase in testing performance across nearly all testing modalities in each subject with the exception of the practical facial nerve exam and the written microtia exam Resident feedback was measured on a Likert scale from 0 (very poor) to 10 (excellent). Feedback was positive with average scores for each component of the module ranging from 8.9 to 9.8. Highest scores were given to simulation workshops
Cervenka et al. (2020)[84]	University of California, Davis, Sacramento, USA	Cross-sectional study, one-day camp Data being reported is from the camp in August of 2016 and 2017	Prior procedural experience of PGY-1 and PGY-2 residents Participant confidence before and after the camp Station efficacy	Trainees showed a statistically significant increase in confidence levels for all task trainer stations All stations had an efficacy Likert score average of 4 “very effective” or 5 “most effective.” Peritonsillar abscess, auricular hematoma, and lateral canthotomy stations had the greatest magnitude of change with 1.4, 1.7, and 1.6 units respectively

*PTA*: peritonsillar abscess, *RCT*: randomized controlled trial, *OHNS*: otolaryngology–head and neck surgery, *MCQ*: multiple choice questions

**Table 2:** Learning objectives and common task trainers used in OHNS boot camps

Study	Learning objective/curriculum design	Task trainer stations
Washington, USA Group	Needs assessment identified common OHNS: airway, bleeding, and other emergencies as high yield topics Program based on graduated levels of complexity allowing participants to develop a framework to build on acquired skills Learning modules contained specific objectives and skills to be accomplished containing elements of the ACGME competencies Overall objectives of the camp were to: recognize and triage typical OHNS emergencies, perform basic emergency management skills, and communicate effectively with the team Objectives designed to be clear, active, and whenever possible measurable	Bag mask ventilation Tracheal intubation Flexible fiberoptic laryngoscopy Microlaryngoscopy/bronchoscopy Epistaxis Cricothyroidotomy with tracheostomy tube change PTA simulator
Canadian Group	Overall camp objectives are for junior OHNS to perform routine emergency on-call procedures, optimize skills in emergency triage, improve communication and leadership skills in stressful situation Camp pedagogy was to deliver simulation in a non-threatening, controlled environment to facilitate trainees improving procedural skills with immediate debrief and feedback	PTA Post Tonsillectomy bleed Epistaxis Lateral canthotomy Surgical airway (Tracheostomy) Non-surgical airway (bronchoscopy and intubation; pediatric and adult)
UK Group	The objective of the program was for participants to understand the management of key topic areas including infectious airway obstruction, epistaxis, post-operative problems, neck trauma, epistaxis, blocked tracheostomy, airway foreign body, and flexible nasal endoscopy Goal of camp was to improve trainee’s knowledge base and performance in the management principles for emergency OHNS scenarios systematic assessment and management principles taught in advanced life support and advanced trauma life support. Teaching emphasized systematic ‘ABC’ approach. Structured feedback was designed to facilitate learning after performing tasks and simulations Curriculum designed to cover OHNS emergencies from a generalist perspective Curriculum utilized the systematic assessment and management principles taught in advanced life support and advanced trauma life support	Basic examination and equipment handling in otology Ear examination, microsuction, foreign body removal Epistaxis: nasal cautery, anterior & posterior packs Flexible nasal endoscopy Tracheostomy/laryngectomy care
New York, USA Group (NYU)	Educational design based on three main principles: defining a set of airway skills for competency, developing educational program designed to address said competencies, and evaluate program using objective educational tools Program based on a mixture of lecture, video, and simulation-based training sessions incorporating ACGME core competencies for airway skills	Bag mask ventilation Tracheal intubation Fiberoptic intubation Placement of laryngeal mask airway Rigid bronchoscopy Jet ventilation Tracheostomy Cricothyroidotomy
New York Group (AECM)	Goal of camp to introduce junior OHNS residents to core skills and principles that may equip them to safely and effectively manage common clinical scenarios in a low-risk learning environment Camp objectives designed to: clinical skills, critical thinking, situational awareness, professionalism, and communication Structed debrief and feedback on performance was administered Immediately following completion of simulation	Soft tissue techniques: suturing and knot tying Soft tissue techniques: knot tying Microsurgical technique: myringotomy Microsurgical technique: laryngeal suturing Sinus simulator: sinonasal polypectomy
Ecuador Group	Goal of program was to introduce three novel simulation teaching modules in facial plastic and reconstructive surgery for capacity building in a low-to middle-income country To address the lack of structured forms of teaching and educational modules while assess efficacy	No task trainers utilized
California, USA Group	Goal of camp was to compare confidence levels before and after the course to evaluate the efficacy of each station Aimed at improving judgement, technical, and critical thinking skills to prepare residents for high-stakes scenarios they may encounter	Six stations: Epistaxis Cricothyrotomy/tracheostomy Peritonsillar abscess/auricular hematoma Nasal bone reduction/zygoma reduction/lateral canthotomy/canalicular trauma and probing Local nerve blocks Soft tissue reconstruction

*ABC*: airway, breathing, circulation, *ACGME*: Accreditation Council of Graduate Medical Education, *AECM*: Albert Einstein College of Medicine, *NYU*: New York University, *OHNS*: Otolaryngology–head and neck surgery, *PTA*: peritonsillar abscess

**Table 3:** Boot camp simulators stratified by OHNS subspecialties

Subspecialty	Task
Otology	Otologic examination, microdebridement, myringotomy, foreign body removal
Rhinology	Nasal cauterization, anterior and posterior nasal packing, polypectomy
Laryngology	Microlaryngoscopy, bronchoscopy, laryngeal suturing
General	Physical examination, Flexible nasopharyngoscopy, bag mask ventilation, jet ventilation, intubation, tracheostomy, suturing, knot tying, peritonsillar draining, post tonsillectomy bleeding control, lateral canthotomy, management of retro-orbital hematoma, tracheostomy care, laryngectomy care

**Table 4:** Common didactic sessions and simulation scenarios in OHNS boot camps

Study	Didactic Sessions	Simulation Scenarios and Feedback
Washington, USA Group	No formal lectures Faculty demonstration prior to each skill station Faculty led case-based exercise exploring common OHNS call scenarios with discussion facilitated by electronic audience response systems	Two team simulation scenarios: Hematoma with airway obstruction after thyroid surgery Angioedema resulting in airway obstruction Faculty-led debrief sessions immediately after simulation designed to address communication, teamwork, decision making, and technical skills
Canadian Group	No formal lectures 1-h task trainer exercises were provided with faculty supervision and instruction if necessary Interactive panel discussion on 16 common emergency clinical scenarios	Two high-fidelity emergency scenario simulations: Post-thyroidectomy hematoma Facial trauma (Facial fracture with difficult oral intubation) Group and individual feedback with faculty post-simulation with video recording
UK Group	Focused lectures in small group organized in two parts: 1. Formal didactic training delivered covering basic systematic assessment of the critically ill patient using ALS and ATLS guidelines 2. Common OHNS topics: airway management, head and neck, rhinology, otology, audiology, pediatric, operations and perioperative care, and radiology For practical skills sessions participants received hands-on instruction from faculty on task trainers	Five teamwork simulation sessions: Airway obstruction Epistaxis and resuscitation Post-tonsillectomy bleed Neck Trauma Post-laryngectomy care Each candidate worked through scenario as either leader or assistant with faculty guidance if needed. Performance videotaped and structured feedback was provided by faculty after sessions
New York, USA Group (NYU)	Formal didactic and video lectures delivered by faculty covering airway evaluation and management with emphasis on difficult airways	Six difficult airway cases designed to test team performance (no details) Team debrief post simulation. Video recorded sessions were randomized and analyzed by three academic OHNS staff on four domains: preparation, clinical reasoning, knowledge, and non-technical skills
New York Group (AECM)	eaching organized into formal didactic sessions and technical skills development Ten, two-hour didactic lectures were offered by attending physicians which covered: introduction to the operating room and basic instruments, flexible laryngoscopy, bronchoscopy, tracheostomy, epistaxis management, laser safety, and subspecialty specific orientations (head and neck, rhinology, and otology)	Eight total simulations falling in to three categories: *Airway simulation.* Scenarios included: angioedema, laryngospasm, trismus, and oropharyngeal bleeding *Epistaxis and bleeding neck simulation.* Scenarios included: anterior nasal bleed, posterior nasal bleed, expanding hematoma *Team based simulation scenarios*. Scenarios included: dislodged tracheostomy tube, post-obstructive pulmonary edema, postoperative stroke, postoperative safe handoff, malignant hyperthermia, epiglottitis, and loss of airway Faculty observed trainees during simulation for demonstration of clinical skills, critical thinking, situational awareness, professionalism, and effective communication. Follow simulations trainees were debriefed on their performance
Ecuador Group	Formal didactic lectures in part of the first half of each day that covered: a review of relevant anatomy, disease processes, facial analysis, and surgical management for each scenario. The second half of the day was spent in live surgery training Residents were also given a flash drive with reading materials, lectures and videos to review	In part of the first half of the day, time was spent practicing pertinent facial analysis and participating in three simulations: Microtia Nasoseptal deformities Facial paralysis Residents performed while being observed by visiting surgeons and received instruction if necessary. If a resident missed part of the sessions, material was reviewed with them separately. Residents were instructed on proper photo documentation for rhinoplasty as well as intraoperative record keeping with Gunter diagrams
California, USA Group	No formal didactic lectures Used cadaveric task trainers in the morning to teach procedural skills followed by simulation-based curriculum in the afternoon	Simulations used included: Airway fire during tracheostomy Pediatric respiratory code during airway evaluation Dislodged pediatric tracheostomy tube in the ICU Angioedema in the emergency department with the inability to intubate or ventilate The task trainers and simulations were run by faculty from the participating institutions

*OHNS*: Otolaryngology–head and neck surgery, *ALS*: advanced life support, *ATLS*: advanced trauma life support

**Table 5:** Common resources utilized in OHNS boot camps

Study	Resources
Washington, USA Group	1. Basic and advanced airway task trainers: adult simulator (SimMan® and AirSim® multi trainers by Laerdal), pediatric simulator (pediatric HAL by Gaurmard), infant simulator (infant and AirSim baby trainer by Laerdal) 2. Epistaxis task trainer: adult airway mannequin with intravenous tubing place within nasal cavity 3. Surgical airway task trainer: fresh porcine larynx 4. PTA task trainer: self-constructed uvula, soft pallet and abscess secured within Resusci Anne mannequin face mask 5. Simulation: SimMan 3 G high fidelity adult-human patient simulator (Laerdal, Wappinger Falls, NY)
Canadian Group	1. Basic and advance airway task trainers: surgical airway stations using porcine model. Surgical airway using combination of pediatric and adult airway models 2. PTA and post-tonsillectomy bleed task trainers: high fidelity cadaveric simulators fresh head and neck cadaveric material. IV tubing containing artificial blood and simulator ‘pus pocket’ surgically placed in anatomical position 3. Surgical airway task trainer: fresh porcine models 4. Simulation: SimMan high fidelity adult-human patient simulator (Laerdal, Wappinger Falls, NY)
UK Group	1. Task trainers: authors do not mention resources 2. Epiglottitis simulation: Laerdal Airway Management Trainer (Laerdal Medical, Stavanger, Norway) 3. Epistaxis simulation: nasal cavity model BIX-LV17 (Chinon Ind., Shanghai, China)
New York, USA Group (NYU)	1. Basic and advanced airway task trainers: pediatric and adult airways (Laerdal, Inc., Wappingers Falls, NY) 2. Surgical airway: cadaveric tracheotomy and cricothyroidotomy. Surgical airway task trainers (Laerdal, Inc., Wappingers Falls, NY) 3. Video lectures: “Management of the Difficult Airway” (Cook Critical Care Division, Cook Inc., Bloomington, IN), and “Adult Airway Management Principles and Techniques” (Silver Platter Education Inc., Newton, MA) 4. Simulation: high-fidelity mannequins used for endoscopy and epistaxis (no details given)
New York Group (AECM)	1. Basic and advanced airway task trainers: no mention of simulators used for adult and pediatric simulations 2. Suturing and knot tying task trainer: traditional pig foot model 3. Microsurgical techniques task trainer (myringotomy and laryngeal suturing): faculty designed simulators (no mention of exact simulator set up) 4. Sinonasal polyps task trainer: simulator using bell peppers and seeds for sinonasal polyps
Ecuador Group	1. Authors mentioned the use of a synthetic rib to plan, carve and assemble an auricular framework in the microtia simulation 2. Novel nasal model stimulator to perform septoplasty, carving and placement of columellar strut grafts, spreader grafts, tip grafts, and for practicing placing a nasal splint 3. Pigs’ feet were used during the facial paralysis workshop on the third day for a suturing workshop to address soft tissue handling deficiencies noted during live surgeries in the previous days
California, USA Group	1. Epistaxis model: tubing directly in the frontal outflow tract through a trephination. Additionally, nasal endoscopy was performed following packing placement 2. Nasal bone/zygoma fracture model: narrow mallet or osteotome to elicit a simple fracture pattern 3. Soft tissue reconstruction station: cadaver heads with soft tissue defects 4. Local nerve blocks station: two cadaveric heads with isolated supraorbital, infraorbital, and mental nerves. Used in combination with a preserved skull to teach the course of the sensory nerves and landmarks 5. Airway fire during tracheostomy, pediatric respiratory code during airway evaluation, dislodged pediatric tracheostomy tube in the ICU, and angioedema in emergency department with inability to intubate or ventilate—SimMan and SimBaby models (Laerdal Medical, Wappingers Falls, NY) 6. Airway exercises station: eight pediatric and adult mannequins 7. Assembly and foreign body extraction: used bronchoscopes and a KARL STORZ tele pack 8. Facial trauma station: Synthes® plating modules and composite skulls

Boot camps were analyzed for their course objectives, outcomes assessed, and overall study conclusions (Table 1). The earliest boot camp identified was in 2011, where Georgetown University (Washington, DC, United States of America) hosted the inaugural training course for junior trainees. This program established the standard to which subsequent boot camps developed their curricula [87]. Thirteen of the sixteen studies described one-day courses, while the remaining three were longitudinal in design, taking place over one- to six-months. All camps incorporated technical skills stations, simulation sessions, and didactic teaching surrounding common OHNS emergencies and consultation requests. Overall, these sixteen studies could more easily be organized into seven international boot camps with their associated academic centres (Tables 2, 4 and 5). Boot camps were based at the University of Georgetown (USA), New York University (USA), Albert Einstein College of Medicine (USA), University of California, Davis (USA), Western University (Canada), University of Cambridge (United Kingdom), and Hospital Un Canto a la Vida (Ecuador).

Most boot camps specifically stated their objectives. Common themes included recognizing and triaging common OHNS emergencies, performing critical basic procedural skills, communicating within a team, and knowing when to call for help. Several task trainers and simulators used for the development of specific procedural skills are listed in Table 2 and categorized by subspecialty in Table 3. The most common simulation scenarios included management of post-surgical and oropharyngeal bleeding (57%), acute airway obstruction from angioedema (43%), and facial/neck trauma (29%). The most common task trainers were surgical airway (71%), epistaxis (57%), peritonsillar abscess drainage (43%), and bag mask ventilation with tracheal intubation (29%). Taking this together, skills stations could be categorized into either 1) basic airway control or 2) special skills. Basic airway control stations include bag mask ventilation, intubation, and surgical airway simulation. Special skill stations include bronchoscopy, peritonsillar abscess drainage, epistaxis and post-tonsillectomy hemorrhage control. Using this terminology allows boot camps to develop goal-oriented simulation stations with thoughtful and explicitly stated objectives.

With respect to each boot camp’s educational frameworks, all courses incorporated some elements of didactic and simulation sessions (Table 4). Didactic sessions involved common OHNS on-call scenarios, emergency situations, operative skills, and perioperative care of the post-surgical patient. Simulation sessions were predominantly focused on acute and subacute OHNS presentations including airway obstruction, epistaxis, and trauma. OHNS simulation resources can be subdivided into physical task trainers to virtual reality platforms [17]. Physical task trainers including mannequin, animal, and cadaveric simulators are often employed (Table 5).

Our synthesis of the data demonstrated that participation in introductory boot camps appears to improve trainee confidence [16, 84, 87, 93], immediate knowledge acquisition [82, 85, 92, 93], and immediate improvement in procedural skills [83, 91] (Table 1). Studies utilizing prospective cohorts and randomized controlled trials (RCTs) revealed an improvement in immediate didactic knowledge (as demonstrated by multiple choice examination), technical skills (based on blinded faculty assessment), and self-perceived confidence which was maintained up to 6 months [82, 87, 91, 93]. In a head-to-head RCT comparing simulation versus traditional didactic learning methods, junior trainees randomized to the simulation arm performed significantly better in both epistaxis and epiglottitis scenarios scored individually by a blinded expert surgeon. Additionally, participants randomized to the simulation group had an improved perception of education and were more likely to make positive recommendations to their colleagues [91].

This study is the first scoping review in OHNS boot camps for junior resident learners. Through our analysis, we have gained valuable insight into the variability of practices around the world. In Table 6 and Table 7, we have summarized our interpreted pros and cons of various boot camps features and developed suggestions for successfully implementing an OHNS surgical boot camp for junior residents.

**Table 6:** Pros and Cons of various boot camp features

Boot camp Feature		Pro	Con
Format	One to Seven-day camp	Ease in set up/execution; less time away from clinical activities	Less time for learning consolidation
	Four-week camp	Additional time in camp may aid in knowledge retention and support better connection from theory to practice	No evidence for long-term benefits; more labour intensive; more time away from clinical activities
Participants	PGY-1 (interns, R1)	Welcoming to profession; perceived ease of transition to residency[103]	None identified
	PGY-2 (R2)	Added expertise may allow for better refinement of skills	None identified
Instructors	OHNS consultants	Ease of organization	None identified
	Multidisciplinary staff (anesthesia, thoracic surgery, emergency medicine)	Added expertise; emphasis on interdisciplinary communication	More complexities in scheduling
Curriculum Design	Didactic- based	Ease in design; improved knowledge retention and comprehension post course[82]	Less interactive; less desired by residents
	Simulation	Surgical learning styles prefer active experimentation[15]; improved resident perceived confidence, competency, and performance[1, 82, 85, 87, 91]; improved learner experience; value in teamwork/collaboration[94]	More costly; more resource intensive

*OHNS*: Otolaryngology–Head and Neck Surgery

**Table 7:** Keys to success for OHNS boot camps.

Boot camp Feature	Suggestions
Format	One to Seven-day camp
Participants	PGY-1 or PGY-2 (junior learners)
Instructors	Multidisciplinary instructors (combined OHNS/Anesthesia/Emergency Medicine)
Curriculum: Boot camp objectives	1. Recognize and triage typical OHNS emergencies: airway obstruction and management (infectious obstruction, foreign body, airway bleeding), post-operative bleeding, epistaxis, post-operative medical complications, neck trauma, blocked tracheostomy, and flexible nasal endoscopy 2. Use systematic assessment and management principles taught through ALS and ATLS 3. Perform basic emergency management skills 4. Communicate effectively with the team
Curriculum: Content	Didactic Component Traditional lecture styles focused on approach and management of typical OHNS emergencies (as above) Task trainer stations Airway: BMV, tracheal intubation, microlaryngoscopy/bronchoscopy, flexible fiberoptic laryngoscopy Surgical techniques and care: basics of surgical instruments, cricothyroidotomy, tracheostomy, tracheostomy tube change Presentation specific management: epistaxis, post tonsillectomy bleed, PTA High yield simulation stations OHNS-specific simulation: Airway obstruction (post thyroidectomy hematoma, infectious angioedema), epistaxis, post tonsillectomy bleed General team-based simulation: postoperative safe handoff, post-operative medical complications (post-obstructive pulmonary edema, post-operative stroke)
Feedback	Facilitation of a safe learning environment with emphasis on resident experience Structured written feedback Preparation (assessment of situation), clinical reasoning, knowledge, technical skills^82^ (see Amin et al.) Simulation feedback Structured debrief and feedback on performance immediately post session
Beyond Boot camp	Base boot camp within other welcoming to the profession activities/institutional rituals (welcome Barbeque, resident retreat etc.)

Suggested boot camp features

*ALS*: advanced life support, *ATLS*: advanced trauma life support, *OHNS*: otolaryngology–head and neck surgery, *PTA*: peritonsillar abscess

One-to-seven-day camps for junior learners provide an optimal balance of relative ease in camp set up and execution with less time away from clinical activities for learners. Multidisciplinary staff including faculty from anesthesia, emergency medicine, thoracic surgery along with OHNS may provide added expertise and allow for more focus on interdisciplinary teamwork which is integral for trainee development. Didactic-based curriculum leads to improvements in knowledge retention and comprehension post course [82] while simulation improves confidence, competence, skill performance, and adds value to the learners’ overall experience with specific emphasis on teamwork / collaboration [1, 82, 85, 87, 91, 94]. Therefore, a curriculum with both didactic and simulation-based learning is advised. Learner feedback should be facilitated in a safe learning environment with emphasis on resident experience with combination of structured written and oral debriefing sessions after simulation (Tables 6 and 7).

## Discussion

Intensive crash courses for residents and fellows have existed in OHNS for numerous years employing simulation to enhance specific aspects of training such as functional endoscopic sinus surgery, removal of foreign bodies, or management of facial trauma [26, 32, 77, 78]. Contrastingly, the concept of an introductory “boot camp” style training course for incoming OHNS trainees emphasizing fundamental skills is a recent occurrence.

As the first published modern-day boot camp for junior OHNS trainees, the Georgetown University boot camp began as a simple, simulation-based one-day emergency course. This has become popularized across the world since its inception in 2011 [87]. Many institutions have adopted similar boot camp style courses for junior trainees with mirroring objectives and content throughout the United States, Canada, and the United Kingdom. Several themes of the modern-day boot camp include the use of simulation, interdisciplinary faculty and trainees (anaesthesia, emergency medicine, family medicine, and pediatrics), and the use of validated educational frameworks for curriculum design (Kolb learning style theory and needs assessment models).

Simulation is an educational approach that enables learners to encounter components of the clinical interactions while enabling educators to provide education and simultaneous assessment in a standardized environment [17, 95]. Widely adopted across various industries, simulation as a training adjunct has become a staple in aerospace and military training, whereas its adoption in medical education has been comparatively slow [96]. In 2012, the Accreditation Council for Graduate Medical Education (ACGME) recognized simulation as a means of evaluating resident performance for various “educational milestones,” in its shift towards competency-based medical education [97, 98]. In OHNS, trainee knowledge and procedural skill were evaluated via cadaveric dissection, temporal bone drilling, and surgical simulator labs [99]. A recent national survey of American OHNS residency programs demonstrated that nearly two-thirds of programs incorporated simulation modalities into curricula [100]. When assessing the Canadian landscape in OHNS programs, 30.8% actively use some form of VR training simulator that 90.9% of program directors felt would be a fair and effective means for evaluation [101]. Given the importance of simulation training in OHNS, many boot camps utilize this method to help junior trainees develop critical skills in a controlled environment.

In this scoping review, all seven boot camps used simulation as the curriculum core through simulation scenarios and specific task trainers. The most common simulation scenarios included management of post-surgical and oropharyngeal bleeding (57%), acute airway obstruction from angioedema (43%), and facial/neck trauma (29%). The most common task trainers were surgical airway (71%), epistaxis (57%), peritonsillar abscess drainage (43%), and bag mask ventilation with tracheal intubation (29%). High fidelity cadaveric and mannequin-based task trainers for task specific procedures appear to be the current trend. All studies that used high fidelity simulation scenarios used the Laerdal (Wappinger Falls, NY) SimMan® adult simulator. SimMan® offers a highly realistic training model with real time neurological and physiological function.

Despite some of the diversity in task trainers and simulations used across the world, the principal theme in all boot camp curricula appeared to be management of emergency situations and on-call scenarios. The goal was to have junior trainees leave the camp equipped with the skillset to identify and triage acute emergencies, perform basic minor airway procedures, and communicate and activate emergency protocols. We noted that trainee participation in introductory boot camps appears to improve their confidence, immediate knowledge acquisition, and immediate improvement in procedural skills in comparison to traditional didactic methods of learning [82, 87, 91, 93]. Simulation learning also improved performance significantly in epistaxis and epiglottis scenarios, improved perception of education and increased the likelihood of making positive recommendations to colleagues when compared to traditional didactic learning methods [91]. The large heterogeneity of the studies included in this review precludes meta-analysis. However, the role of this scoping review was to examine OHNS boot camps more descriptively around the world. Here we have identified a trend in the literature suggesting positive outcomes for trainees that participate in introductory boot camps for their overall clinical and psychosocial development as an early trainee.

Despite strongly positive outcomes from boot camps and simulation training, criticisms of the lack of evidence to suggest long-term retention exist [31, 67]. Three studies demonstrated that perceived confidence in procedural tasks and knowledge lasted up to 2- 6 months [87, 93, 102]. However, neither long-term knowledge retention nor procedural competency has been assessed among OHNS trainees. Also, according to a survey of OHNS residency program directors in the United States and Puerto Rico, there are several barriers that exist which prevent participation in boot camps and simulation training [67]. Some of these include cost, lack of local access, lack of interest, and scheduling difficulties [67]. This suggests making boot camp programs more widely available, having partially subsidized costs, and more data on their short- and long-term benefits could address the hesitancy that some program directors have.

Although boot camps are typically delivered at the beginning of OHNS programs because they are introductory, consensus on when they should be offered is lacking. When surveying American OHNS program directors, a slight majority felt boot camps should be offered within the first few months of residency [67]. Interestingly, simulation training programs have been shown to be effective in all postgraduate years, with knowledge and skills acquisition demonstrated across all training levels [31]. Several other studies have evaluated the effectiveness of OHNS boot camps for medical students and suggest that boot camps may aid with the transition to residency as they all reported improved knowledge, confidence, and clinical performance after completion of the course [27, 33, 38, 62]. Taking these pieces of evidence together, it seems that the surgical boot camp style of education delivery at any level is beneficial in the short-term of less than six months. The lasting effects, however, remain uncertain and future investigations should examine the long-term retention of knowledge, confidence, and technical skill.

## Conclusion

Boot camp style training programs for junior OHNS are becoming widely adopted across the world. Fuelled by the utilization of simulation technology to deliver time-effective education for common OHNS emergencies, these programs embrace the educational shift towards competency-based accreditation standards for residency programs. A number of studies have justified this form of education to improve trainee’s performance, confidence, and skill in the short term. However, current literature has failed to examine a number of important long-term outcomes. Future studies that examine the effect of OHNS boot camps on long term outcomes will play a critical role justifying widespread adoption of boot camps for resident education.

## Data Availability

Not applicable. No datasets were generated or analyzed during this study.
